# Octacosanol and policosanol prevent high-fat diet-induced obesity and metabolic disorders by activating brown adipose tissue and improving liver metabolism

**DOI:** 10.1038/s41598-019-41631-1

**Published:** 2019-03-26

**Authors:** Rahul Sharma, Takashi Matsuzaka, Mahesh K. Kaushik, Takehito Sugasawa, Hiroshi Ohno, Yunong Wang, Kaori Motomura, Takuya Shimura, Yuka Okajima, Yuhei Mizunoe, Yang Ma, Zahara M. Saber, Hitoshi Iwasaki, Shigeru Yatoh, Hiroaki Suzuki, Yuichi Aita, Song-iee Han, Yoshinori Takeuchi, Naoya Yahagi, Takafumi Miyamoto, Motohiro Sekiya, Yoshimi Nakagawa, Hitoshi Shimano

**Affiliations:** 1 Department of Internal Medicine (Endocrinology and Metabolism), Faculty of Medicine, University of Tsukuba, 1-1-1 Tennodai, Tsukuba, Ibaraki 305-8575 Japan; 2 Transborder Medical Research Center, University of Tsukuba, 1-1-1 Tennodai, Tsukuba, Ibaraki 305-8575 Japan; 3 International Institute for Integrative Sleep Medicine (WPI-IIIS), University of Tsukuba, 1-1-1 Tennodai, Tsukuba, Ibaraki 305-8575 Japan; 4 Life Science Center for Survival Dynamics, Tsukuba Advanced Research Alliance (TARA), University of Tsukuba, 1-1-1 Tennodai, Tsukuba, Ibaraki 305-8575 Japan; 5 AMED-CREST, Japan Agency for Medical Research and Development (AMED), 1-7-1, Ohte-machi, Chiyoda-ku, Tokyo 100-0004 Japan

## Abstract

Brown adipose tissue (BAT) is an attractive therapeutic target for treating obesity and metabolic diseases. Octacosanol is the main component of policosanol, a mixture of very long chain aliphatic alcohols obtained from plants. The current study aimed to investigate the effect of octacosanol and policosanol on high-fat diet (HFD)-induced obesity. Mice were fed on chow, or HFD, with or without octacosanol or policosanol treatment for four weeks. HFD-fed mice showed significantly higher body weight and body fat compared with chow-fed mice. However, mice fed on HFD treated with octacosanol or policosanol (HFDo/p) showed lower body weight gain, body fat gain, insulin resistance and hepatic lipid content. Lower body fat gain after octacosanol or policosanol was associated with increased BAT activity, reduced expression of genes involved in lipogenesis and cholesterol uptake in the liver, and amelioration of white adipose tissue (WAT) inflammation. Moreover, octacosanol and policosanol significantly increased the expression of *Ffar4*, a gene encoding polyunsaturated fatty acid receptor, which activates BAT thermogenesis. Together, these results suggest that octacosanol and policosanol ameliorate diet-induced obesity and metabolic disorders by increasing BAT activity and improving hepatic lipid metabolism. Thus, these lipids represent promising therapeutic targets for the prevention and treatment of obesity and obesity-related metabolic disorders.

## Introduction

Obesity, which results from a chronic imbalance between energy intake and expenditure, is a strong risk factor for several metabolic diseases, such as type 2 diabetes mellitus, hyperlipidaemia, atherosclerosis and nonalcoholic fatty liver disease (NALFD). Several current anti-obesity drugs that aim to reduce food intake cause severe psychiatric or cardiovascular side effects^1,2^. Therefore, alternative safety therapeutic strategies, such as enhancing energy expenditure, are urgently needed to combat obesity.

White adipose tissue (WAT) is essential for the storage of excess energy as triglycerides (TGs), while brown adipose tissue (BAT) dissipates energy in the form of heat by uncoupling mitochondrial respiratory chain and ATP biosynthesis^3^. Active BAT has been found in adult humans, which is believed to play an essential role in metabolic balance in the body^4,5^ and has been emerging as a promising target to treat obesity and diabetes through its energy expenditure pathway^6,7^. Some human studies have suggested that obesity might be associated with a decreased volume of BAT^8,9^, and animal studies have also shown that absence of BAT activity could aggravate obesity^10–12^. Recent studies have revealed the ability of specific WAT depots to activate thermogenesis upon exposure to cold and hormonal stimuli^13,14^. A subpopulation of cells in inguinal WAT (iWAT), known as “beige” cells, expresses uncoupling protein-1 (UCP-1) and carries out thermogenesis. Thus, promoting the development and function of brown and beige fat appears as an attractive treatment for obesity and obesity-related metabolic diseases.

Policosanol is a mixture of very-long-chain saturated fatty alcohols purified from natural sources such as rice bran, wheat, sugarcane and bees wax, whose main component is octacosanol (CH_3_(CH_2_)_26_CH_2_OH)^15,16^. Previous studies have demonstrated that octacosanol suppresses HFD-induced increase in peripheral adipose tissue weight^17^, lowers blood cholesterol^17–21^, suppresses platelet aggregation^22,23^, reduces inflammation^24–26^, increases athletic performance^27–30^, protects cells^31^, alleviates stress and restores stress-affected sleep^32^. Thus, octacosanol (policosanol) could be used as a drug or food supplement for treating metabolic diseases without any side effects.

A study investigating the biodistribution and metabolism of radiolabeled octacosanol in rats after oral administration has demonstrated higher octacosanol radioactivity in the liver, muscle and adipose tissue, especially BAT^33^. Moreover, previous studies have demonstrated that shortened saturated fatty acids (FAs; myristic, palmitic and stearic) and unsaturated FAs (oleic, palmitoleic) are formed after oral administration of policosanol to monkeys^34^. These FAs might be utilized for energy expenditure via β-oxidation^35^.

The β-oxidation of FAs is critical for the function of BAT and beige fat and hence thermogenesis^36,37^. Recent studies using experimental models and humans suggest that the long chain saturated FAs, mainly stearic acid (C18:0), regulate mitochondrial function^38,39^. Moreover, the ELOVL family member 6 (Elovl6), a microsomal enzyme responsible for converting C16 FAs into C18 species, has been suggested to regulate BAT thermogenic capacity^40^.

Considering that the longer FAs could be preferred substrates for thermogenesis and the abovementioned effects of octacosanol or policosanol on lipid metabolism, their role in the prevention or treatment of obesity and in the thermogenic function of brown and beige adipocytes has not yet been established. In the present study, we investigated anti-obesity and thermogenic function of octacosanol and policosanol in mice.

## Results

### Octacosanol and policosanol prevent HFD-induced obesity

**Figure 1:**
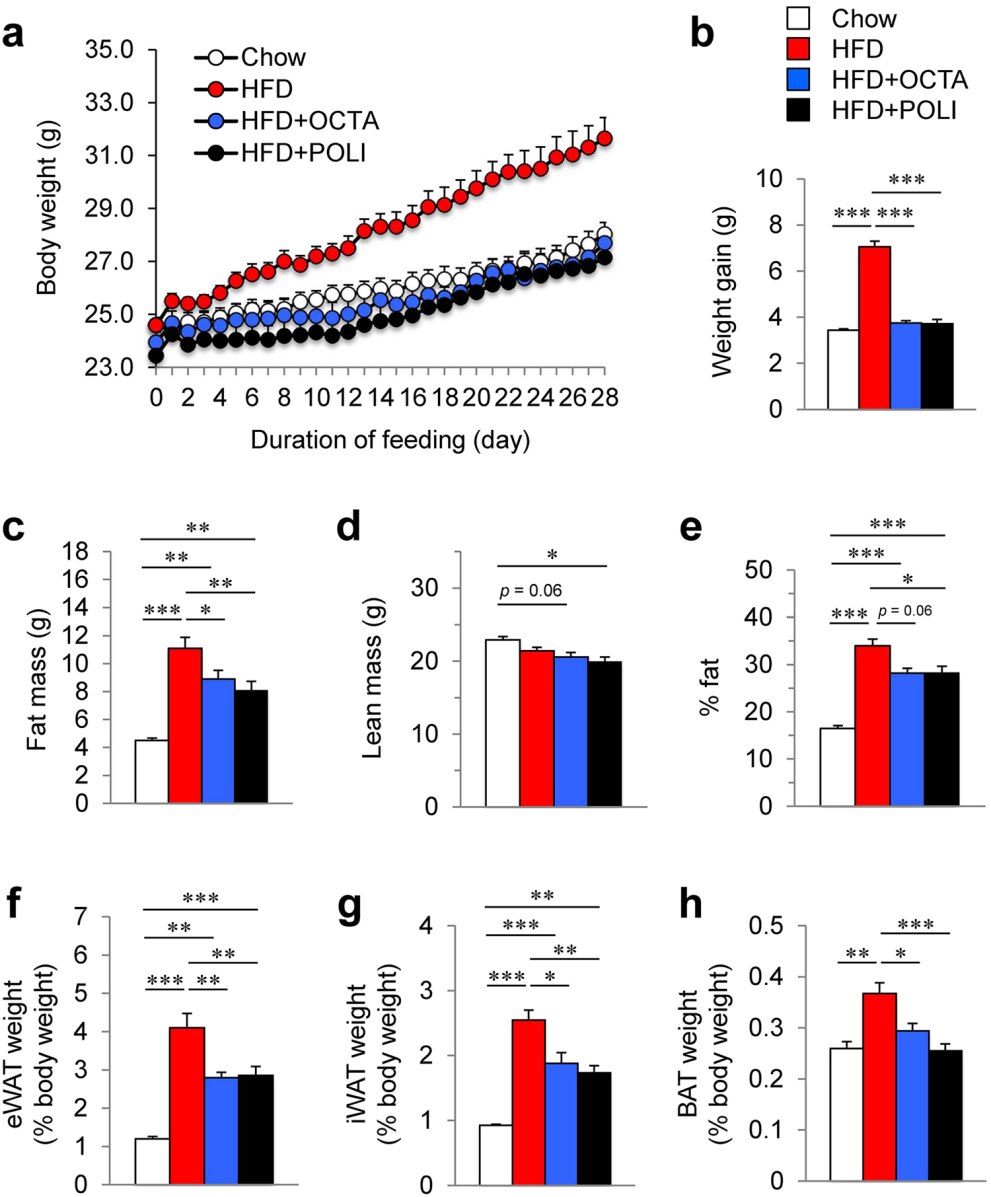
Effect of octacosanol and policosanol on body weight, body composition and fat accumulation in mice fed on chow, high fat diet (HFD) and HFD treated with octacosanol or policosanol. (**a**,**b**) Body weight (**a**) and body weight gain (**b**) of mice fed on chow, HFD and HFD treated with octacosanol or policosanol (60 mg/kg/day) for four weeks (n = 7–8). (**c**–**e**) Changes in fat mass (**c**), lean mass (**d**) and percent fat (**e**) measured by dual-energy X-ray absorptiometry (DEXA) analysis (n = 5). (**f**–**h**) Weight of epididymal white adipose tissue (eWAT) (**f**), inguinal WAT (iWAT) (**g**), brown adipose tissue (BAT) (**h**) of mice fed on chow, HFD and HFD treated with octacosanol or policosanol for four weeks (n = 7–8). Values represent mean ± standard error of mean (SEM). *P < 0.05, **P < 0.01, ***P < 0.001 by using one-way ANOVA followed by scheffe post hoc test.

To investigate the effect of octacosanol and policosanol on the development of obesity, male C57BL/6 mice were fed on normal chow, HFD, or HFDo/p for four weeks. The body weight (BW) of HFD-fed mice was significantly higher than that of chow-fed mice (Fig. 1a). Treatment with octacosanol or policosanol suppressed the HFD-induced BW gain (Fig. 1a,b). The fat mass gain was significantly less in mice fed on HFDo/p compared with HFD-fed mice (Fig. 1c). The lean body mass tended to reduce in mice fed on HFDo/p compared with chow- or HFD-fed mice (Fig. 1d). Total body fat percentage was also significantly lower in mice fed on HFDo/p (Fig. 1e). The weight of epididymal WAT (eWAT) (Fig. 1f), iWAT (Fig. 1g) and BAT (Fig. 1h) was significantly reduced in HFD-fed mice supplemented with octacosanol and policosanol.

### Octacosanol or policosanol treatment of HFD reduces plasma insulin levels

**Figure 2:**
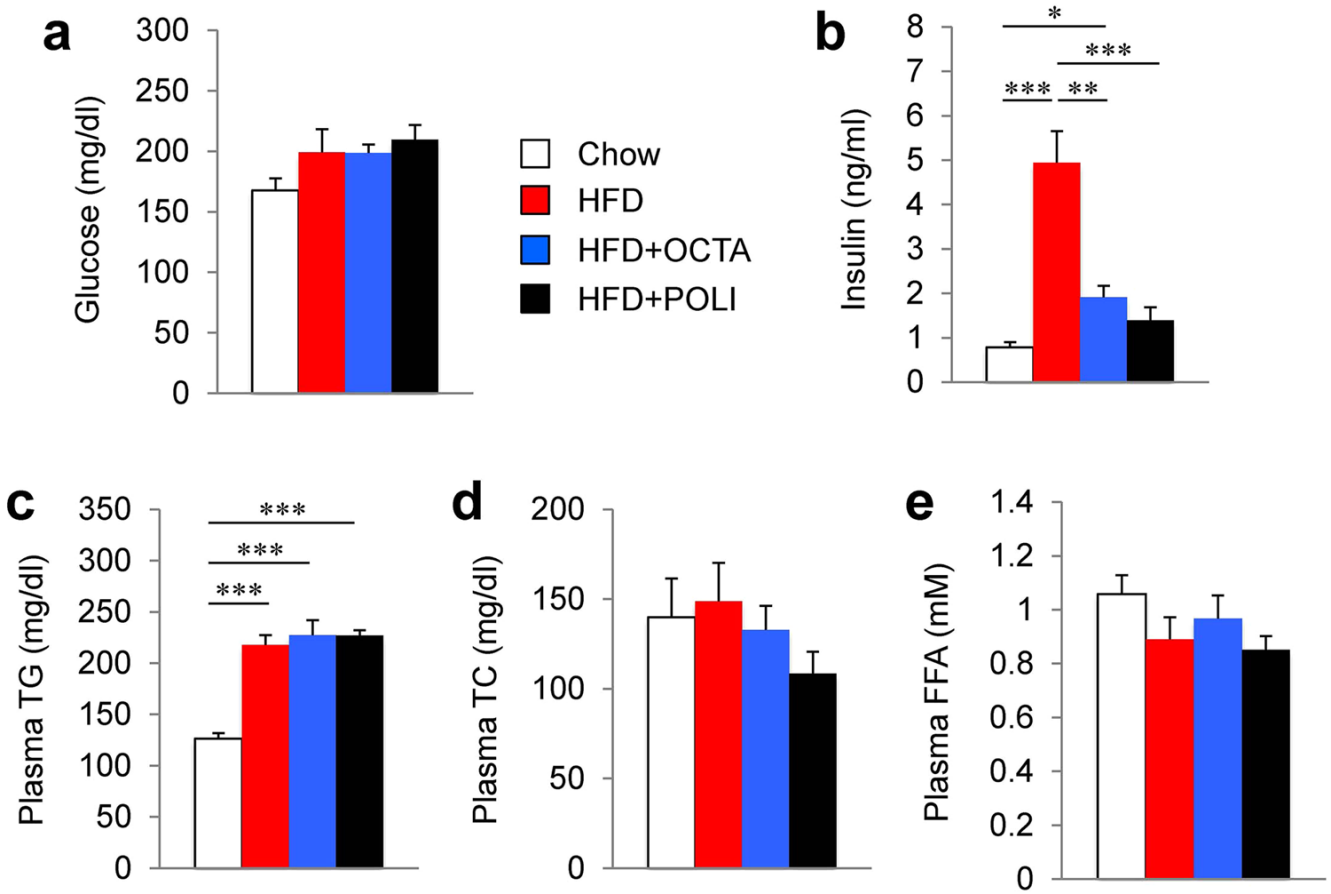
Effect of octacosanol and policosanol on plasma metabolic parameters of mice fed on chow, HFD and HFD treated with octacosanol or policosanol. (**a**–**e**) Concentrations of blood glucose (**a**), plasma insulin (**b**), plasma triglycerides (TGs) (**c**), plasma total cholesterol (TC) (**d**) and plasma free fatty acids (FFA) (**e**) of mice fed on chow, HFD and HFD treated with octacosanol or policosanol for four weeks (n = 5). Values represent mean ± SEM. *P < 0.05, **P < 0.01, ***P < 0.001 by using one-way ANOVA followed by scheffe post hoc test.

Figure 2 compares plasma metabolic parameters in four dietary groups; chow-fed, HFD-fed, HFD treated with octacosanol or policosanol on the 4 h fasted condition. Plasma glucose levels did not show a significant increase in the HFD group compared with the chow group (Fig. 2a). The treatment of HFDo/p did not change plasma glucose levels. Plasma insulin levels were markedly increased in the HFD group (Fig. 2b), demonstrating that HFD causes insulin resistance. Conversely, insulin resistance induced by the HFD was efficiently attenuated by the HFDo/p, as reflected in significantly decreased plasma insulin levels than in HFD-fed mice. Previous studies have shown that octacosanol supplementation reduces serum lipid concentrations in mice^17,18^. However, HFD-fed mice treated with octacosanol or policosanol showed no significant reduction in the plasma levels of TG, total cholesterol (TC), and free fatty acids (FFA) compared with those of HFD-fed mice (Fig. 2c–e).

### Octacosanol and policosanol activate BAT in HFD-fed mice

**Figure 3:**
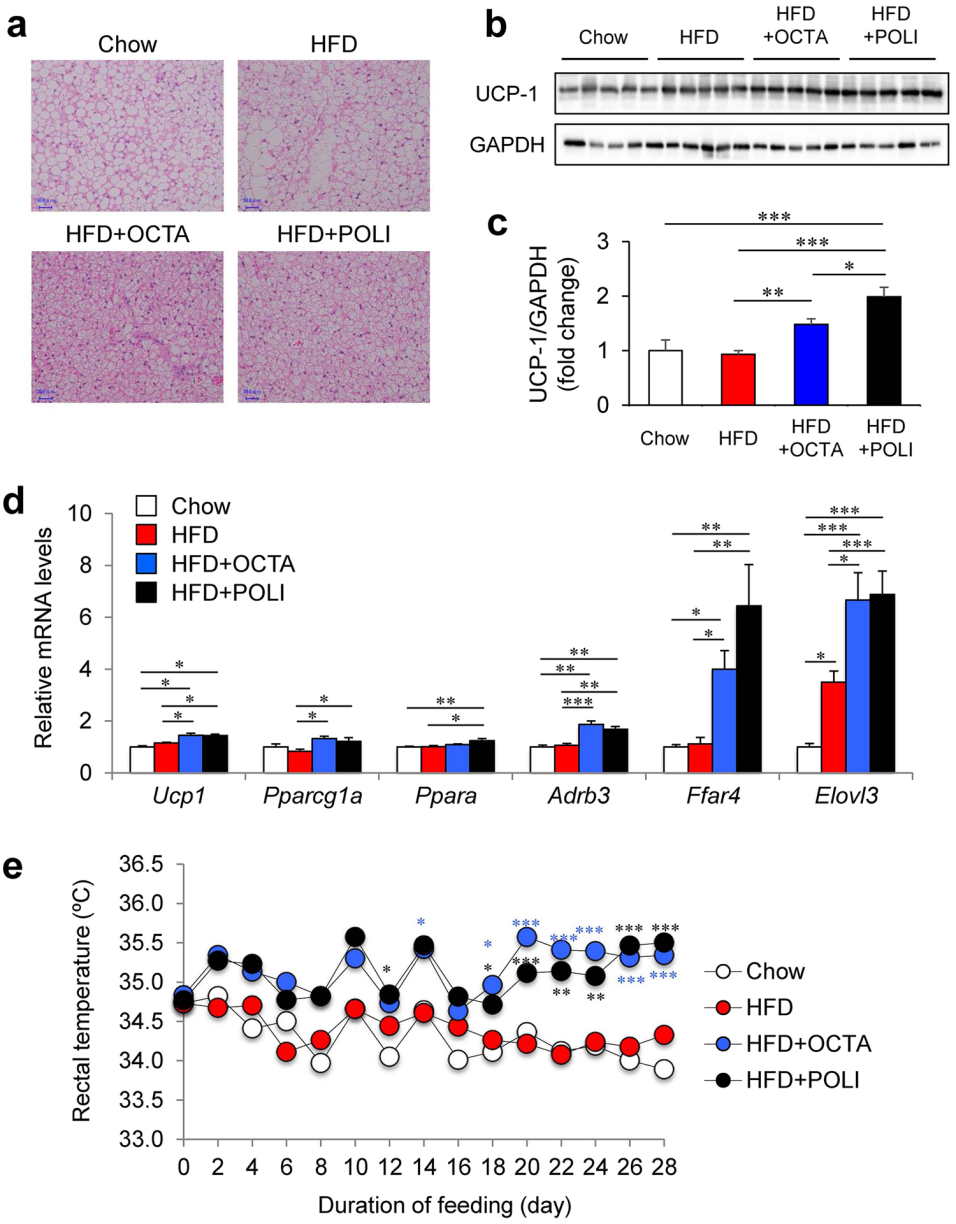
Effect of octacosanol and policosanol on brown adipose tissue (BAT) of mice fed on chow, HFD and HFD treated with octacosanol or policosanol. (**a**) Representative hematoxylin and eosin (H&E)-stained sections of BAT harvested from mice fed on chow, HFD and HFD treated with octacosanol or policosanol for four weeks. (**b**) Western blotting of UCP-1 and GAPDH in BAT (n = 5). GAPDH was used as a loading control. (**c**) Protein expression of UCP-1 determined by densitometry analysis (n = 5). (**d**) Quantitative real-time PCR (qRT-PCR) analysis of genes involved in thermogenesis of BAT in mice fed on chow, HFD and HFD treated with octacosanol or policosanol for four weeks (n = 5–8). (**e**) Rectal temperature of mice fed on chow, HFD and HFD treated with octacosanol or policosanol for four weeks (n = 5). Values represent mean ± SEM. *P < 0.05, **P < 0.01, ***P < 0.001 by using one-way ANOVA followed by scheffe post hoc test.

Previous studies have shown that orally administered octacosanol was distributed to adipose tissue, especially BAT, and is partly oxidized and degraded to FAs through β-oxidation^33–35^. Thus, we investigated the effects of octacosanol and policosanol on BAT (Fig. 3). Histological analysis revealed that the BAT of HFD-fed mice showed markedly enlarged adipocytes compared with that of chow-fed mice (Fig. 3a). However, the HFDo/p prevented HFD-induced enlargement of adipocytes in BAT, indicating reduced lipid accumulation within the tissue probably because they were used more as a metabolic fuel. Western blot analysis showed that protein level of UCP-1 was significantly increased by the HFDo/p (Fig. 3b,c). Moreover, UCP-1 immunohistochemistry further revealed higher levels of UCP-1 in the BAT of HFDo/p-fed mice compared with that of chow- or HFD-fed mice (Supplementary Fig. S1). The expression of thermogenic genes, including *Ucp1*, peroxisome proliferative activated receptor, gamma, co-activator 1α (*Ppargc1a*), β3-adrenergic receptor (*Adrb3*), FFA receptor 4 (*Ffar4*), and *Elovl3*, was significantly increased in BAT of mice fed on HFDo/p (Fig. 3d). Furthermore, mice fed on HFDo/p exhibited a higher core body temperature than chow- or HFD-fed mice (Fig. 3e). These results suggest that octacosanol and policosanol activate BAT activation through the upregulation of thermogenic gene expression.

### Octacosanol and policosanol upregulate genes involved in adaptive thermogenesis in iWAT of HFD fed mice

**Figure 4:**
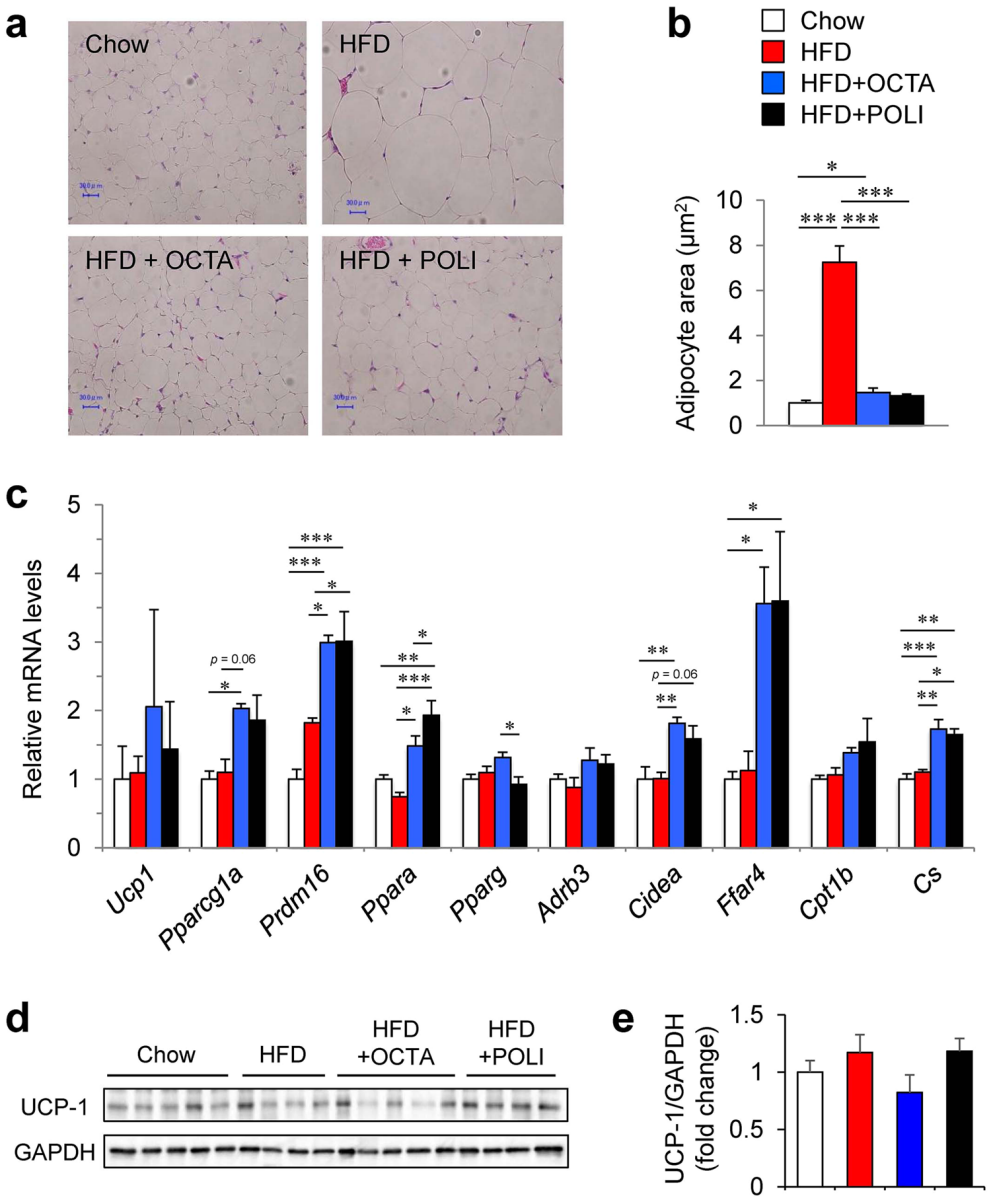
Effect of octacosanol and policosanol on inguinal white adipose tissue (iWAT) of mice fed on chow, HFD and HFD treated with octacosanol or policosanol. (**a**) Representative H&E-stained sections of iWAT harvested from mice fed on chow, HFD and HFD treated with octacosanol or policosanol for four weeks. Scale bar = 30 μm. (**b**) Average adipocyte size in iWAT of mice fed on chow, HFD and HFD treated with octacosanol or policosanol for four weeks (n = 5). (**c**) qRT-PCR analysis of beige fat markers in iWAT harvested from mice fed on chow, HFD and HFD treated with octacosanol or policosanol for four weeks (n = 5–8). (**d**) Western blotting of UCP-1 and GAPDH in iWAT (n = 4–5). GAPDH was used as a loading control. (**e**) Protein expression of UCP-1 determined by densitometry analysis (n = 4–5). Values represent mean ± SEM *P < 0.05, **P < 0.01, ***P < 0.001 by using one-way ANOVA followed by scheffe post hoc test.

Beiging of iWAT has been highlighted as a new possible therapeutic target for obesity, diabetes and lipid metabolic disorders because beiging of iWAT is known to increase energy expenditure and reduce adiposity^3^. Since our results suggest that octacosanol and policosanol activate non-shivering thermogenesis in BAT and reduce body fat, we assessed the effect of octacosanol and policosanol on iWAT. Histological analysis showed large adipocytes in iWAT in HFD-fed mice; however, mice fed on HFDo/p showed significantly smaller adipocytes in iWAT (Fig. 4a,b). Next, we examined whether the diminished adipocyte size in mice fed on HFDo/p could be the result of increased beiging of iWAT. We found that the HFDo/p increased the expression of genes involved in adaptive thermogenesis, such as *Ppargc1a*, PR domain containing 16 (*Prdm16*), *Ppara*, cell death-inducing DNA fragmentation factor, alpha subunit-like effector A (*Cidea*), and *Ffar4*, as well as citrate synthase (*Cs*), a crucial component of the tricarboxylic (TCA) cycle of mitochondria (Fig. 4c). However, we unexpectedly found that the mRNA levels of *Ucp1* and its transcriptional regulator PPAR gamma (*Pparg*) and the protein level of UCP-1 was not increased in the iWAT of HFDo/p-fed mice (Fig. 4c–e). These data suggest that octacosanol and policosanol activate energy combustion but do not fully promote the iWAT beiging in HFD-fed mice.

### Octacosanol and policosanol reduce adipocyte size and lower inflammation in epididymal WAT (eWAT) in HFD-fed mice

**Figure 5:**
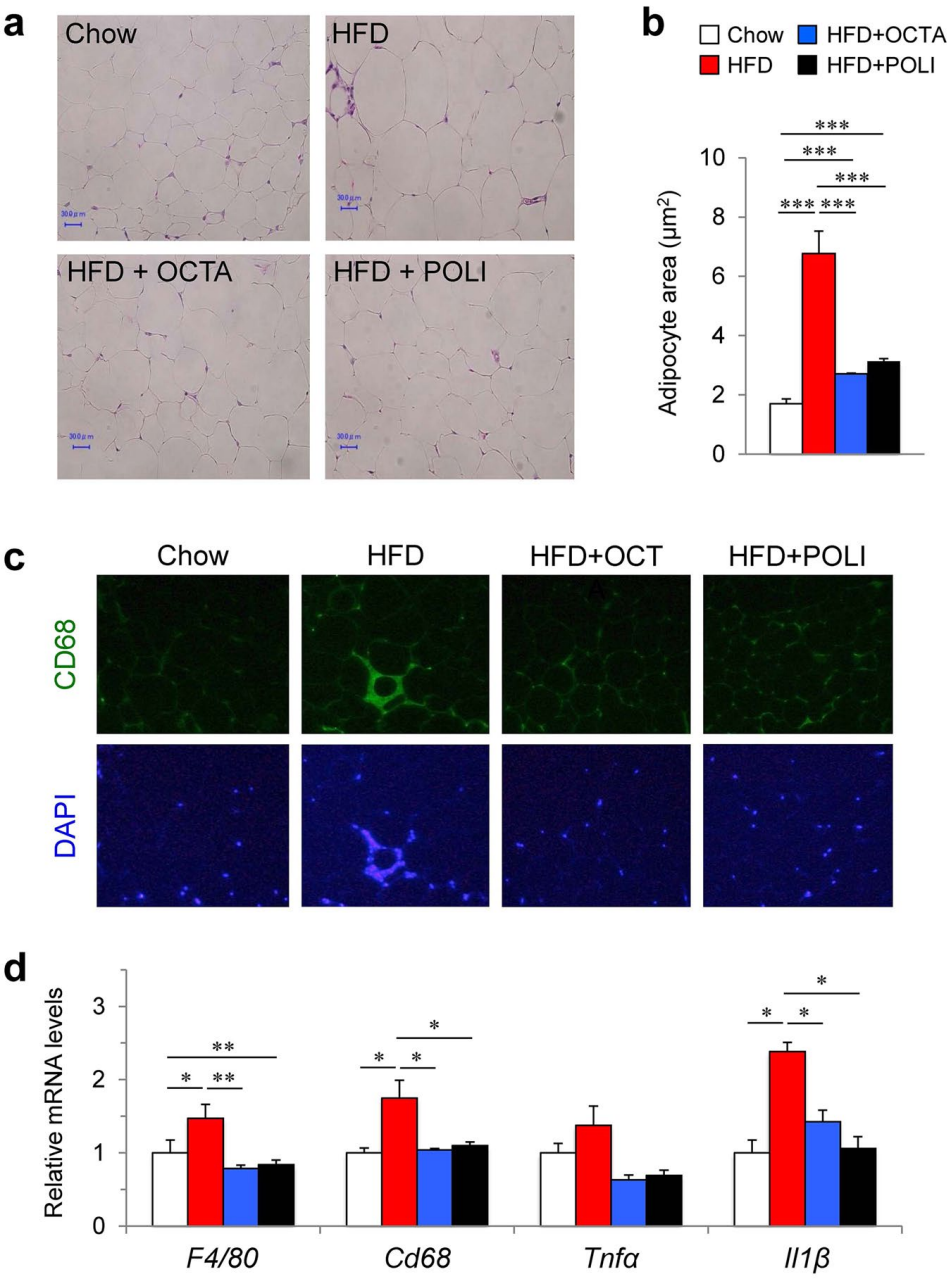
Effect of octacosanol and policosanol on epididymal WAT (eWAT) of mice fed on chow, HFD and HFD treated with octacosanol or policosanol. (**a**) Representative H&E-stained sections of eWAT harvested from mice fed on chow, HFD and HFD treated with octacosanol or policosanol for four weeks. Scale bar = 30 μm. (**b**) Average adipocyte size in eWAT of mice fed on chow, HFD and HFD treated with octacosanol or policosanol for four weeks (n = 5). (**c**) Representative immunohistochemical staining for CD68 in eWAT sections of mice fed on chow, HFD and HFD treated with octacosanol or policosanol for four weeks. (**d**) qRT-PCR analysis of genes involved in the inflammation of iWAT harvested from mice fed on chow, HFD and HFD treated with octacosanol or policosanol for four weeks (n = 5). Values represent mean ± SEM. *P < 0.05, **P < 0.01, ***P < 0.001 by using one-way ANOVA followed by scheffe post hoc test.

We also examined the effect of octacosanol and policosanol on eWAT. Histological analysis showed reduced adipocyte size in eWAT of mice fed on HFD treated with octacosanol or policosanol (Fig. 5a,b). Since octacosanol also possesses anti-inflammatory activity^24–26^, we examined the effects of octacosanol and policosanol on HFD-induced adipose tissue inflammation. In line with the reduced eWAT weight (Fig. 1f), immunohistochemical staining of eWAT sections for CD68, a marker of infiltrated macrophage, revealed that crown-like structures in mice fed on HFDo/p were markedly reduced relative to mice fed on HFD (Fig. 5c). Moreover, the expression of genes associated with inflammation including *F4/80*, Cd68 antigen (*Cd68*), tumor necrosis factor *α* (*Tnfa*) and interleukin 1 β (*Il1b*) was markedly upregulated in the eWAT of mice fed on HFD (Fig. 5d). The induction of these genes was suppressed in mice fed on HFDo/p. These results suggest that, besides preventing fat accumulation in WAT, octacosanol and policosanol also prevent HFD-induced adipose tissue inflammation.

### Octacosanol and policosanol attenuate fatty liver in HFD-fed mice

**Figure 6:**
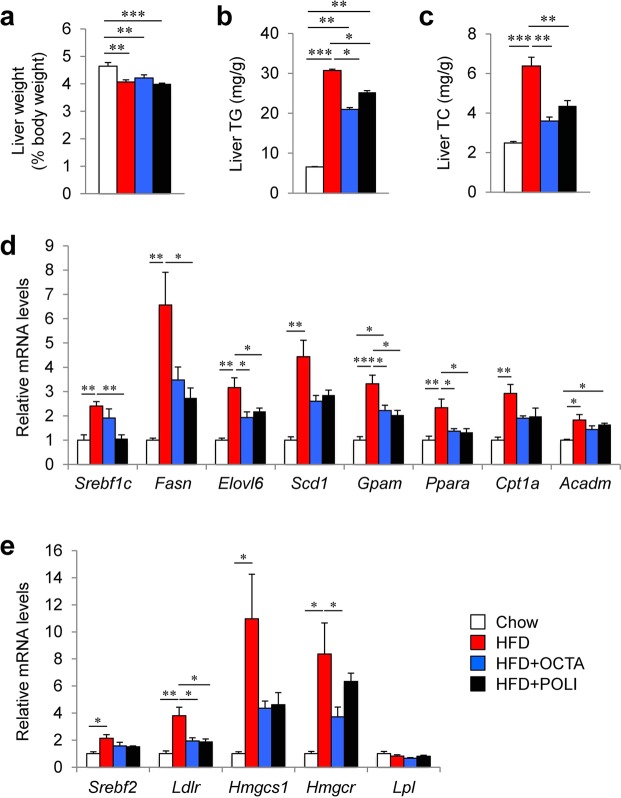
Effect of octacosanol and policosanol on the liver ofmice fed on chow, HFD and HFD treated with octacosanol or policosanol. (**a**–**c**) Liver weight (**a**) and hepatic levels of TG (**b**) and TC (**c**) in mice fed on chow, HFD and HFD treated with octacosanol or policosanol for four weeks (n = 5–8). (**d**,**e**) qRT-PCR analysis of genes involved in fatty acid (FA) metabolism (**d**) and cholesterol biosynthesis (**e**) in livers harvested from mice fed on chow, HFD and HFD treated with octacosanol or policosanol for four weeks (n = 5). Values represent mean ± SEM. *P < 0.05, **P < 0.01, ***P < 0.001 by using one-way ANOVA followed by scheffe post hoc test.

The liver regulates energy metabolism in the body through the dynamic control of glucose and lipid metabolism. Therefore, we investigated the effect of octacosanol and policosanol on the liver. The weight of liver in HFD-fed mice was lower than in chow-fed mice (Fig. 6a); however, HFD markedly increased hepatic levels of TG and TC (Fig. 6b). While the liver weights were similar between HFD-fed mice and HFDo/p-fed mice (Fig. 6a), hepatic levels of TG and TC were significantly lower in the HFDo/p group compared with the HFD group (Fig. 6b,c). These results suggest that octacosanol or policosanol supplementation attenuates the increase in hepatic TG and TC levels caused by HFD. The expression of genes involved in lipogenesis and β-oxidation was analysed by quantitative real-time PCR (qRT-PCR) (Fig. 6d). Expression levels of sterol regulatory element-binding protein-1c (*Srebf1c*) and its downstream genes involved in lipogenesis such as FA synthase (*Fasn*), *Elovl6*, stearoyl-CoA desaturase-1 (*Scd1*) and glycerol-3-phosphate acyltransferase, mitochondrial (*Gpam*) were significantly increased in HFD-fed mice compared with chow-fed mice. Expression levels of *Elovl6* and *Gpam* in mice fed on HFDo/p, and of *Srebf1c* and *Fasn* in mice fed on HFD treated with policosanol were significantly lower than in HFD-fed mice. Expression levels of PPAR alpha (*Ppara*) and its target genes involved in β-oxidation, including carnitine palmitoyltransferase 1a (*Cpt1a*) and acyl-Coenzyme A dehydrogenase, medium chain (*Acadm*), were significantly increased in HFD-fed mice compared with chow-fed mice. HFDo/p significantly reduced *Ppara* expression; it also reduced the expression levels of *Cpt1a* and *Acadm*, although the reduction was non-significant. We also examined the expression levels of genes involved in cholesterol biosynthesis and uptake. Results showed that the treatment of octacosanol or policosanol significantly suppressed HFD-induced increase in the expression of low density lipoprotein receptor (*Ldlr*) (Fig. 6e). Moreover, the treatment of HFDo/p tended to reduce the expression levels of *Srebf2* and 3-hydroxy-3-methylglutaryl-Coenzyme A synthase 1 (*Hmgcs1*), and octacosanol treatment significantly reduced 3-hydroxy-3-methylglutaryl-Coenzyme A reductase (*Hmgcr*) expression. The expression level of lipoprotein lipase (*Lpl*), the rate-limiting enzyme to catalyze the hydrolysis of TG in circulation^41^, is similar in all groups. Collectively, these results suggest that octacosanol and policosanol ameliorate the development of HFD-induced fatty liver by suppressing the biosynthesis of FAs and TGs and uptake of cholesterol by the liver.

### Octacosanol activates BAT and iWAT in normal chow-fed mice

**Figure 7:**
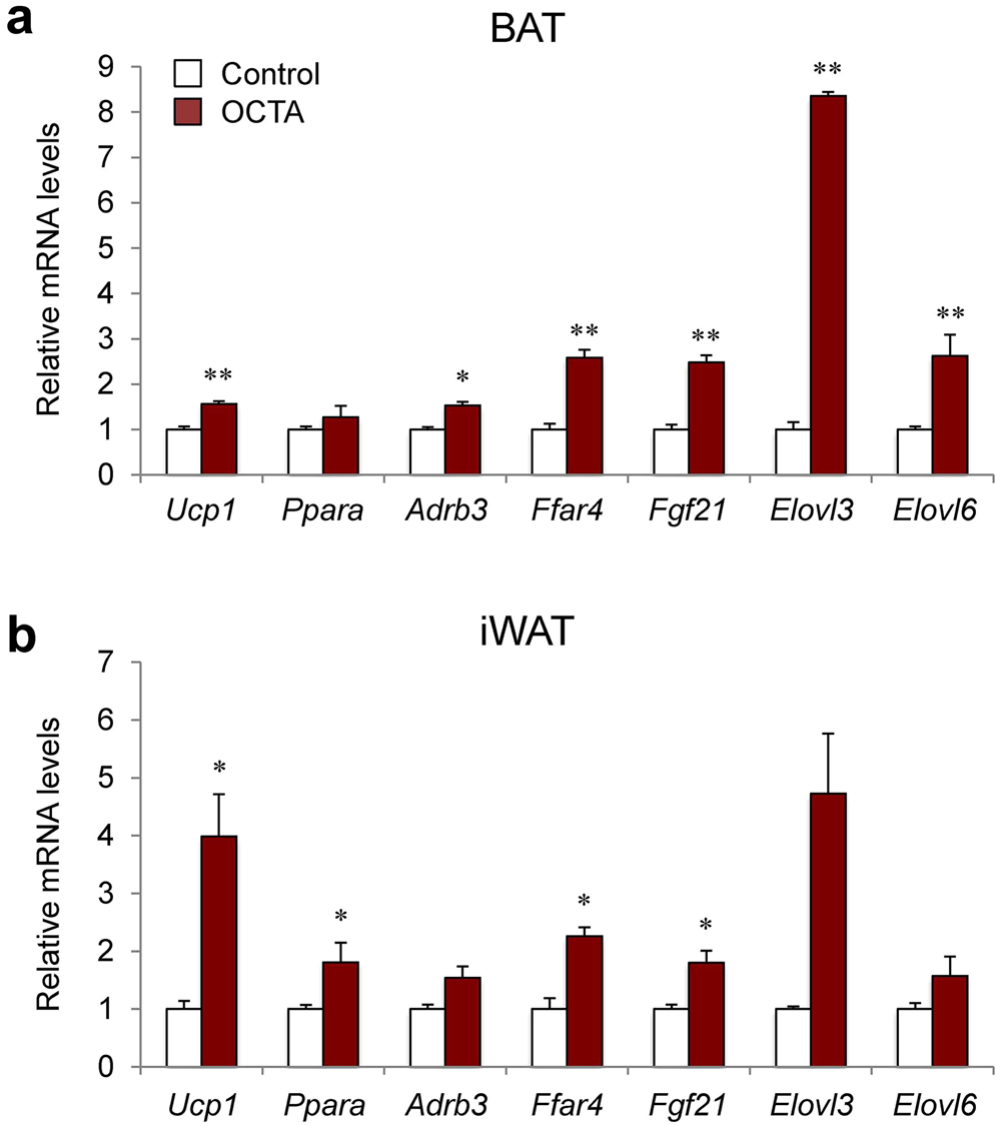
Effect of octacosanol on gene expression in BAT and iWAT harvested from mice fed a chow diet. (**a**,**b**) qRT-PCR analysis of genes involved in thermogenesis and energy expenditure of BAT (**a**) and iWAT (**b**) in mice fed on chow, with or without octacosanol treatment, for seven days (n = 5). Values represent mean ± SEM. *P < 0.05, **P < 0.01, ***P < 0.001 vs. control (vehicle-treated) mice by using student-t-test.

To examine whether HFD is necessary for the effect of octacosanol and policosanol on BAT and iWAT, we investigated the effect of octacosanol on chow-fed mice. Mice fed on standard chow supplemented with octacosanol for seven days showed significantly higher mRNA levels of genes involved in BAT function, including *Ucp1*, *Adrb3*, *Ffar4*, and fibroblast growth factor 21 (*Fgf21*) (Fig. 7a). Similarly, higher mRNA levels of *Ucp1* and other marker genes for adaptive thermogenesis such as *Ppara*, *Ffar4*, and *Fgf21* were observed in the iWAT of mice fed on standard chow treated with octacosanol. Moreover, expression levels of *Elovl6* and *Elovl3*, genes encoding FA elongases involved in the biosynthesis of long chain and very long chain saturated FAs, were significantly increased in BAT and tended to increase in iWAT, respectively, following octacosanol treatment. These data suggest that octacosanol activates BAT and iWAT not only in mice fed on HFD (as shown above) but also in mice fed on chow.

## Discussion

This study aimed to determine whether purified octacosanol and policosanol have beneficial effects on HFD-induced obesity in mice. We showed that both octacosanol and policosanol significantly ameliorated HFD-induced body fat gain and fatty liver. Furthermore, octacosanol and policosanol activated signalling pathways that regulate thermogenesis and enhance energy expenditure in BAT.

Octacosanol or policosanol treatment completely suppressed HFD-induced BW gain. This is presumably due to not only the significant reduction of adipose tissue mass but also the slight reduction of lean mass. The organ(s) that contributes to the reduction of lean mass by the treatment of octacosanol or policosanol is currently unknown. Previous study investigating the biodistribution of radiolabeled octacosanol in rats after oral administration has demonstrated moderate octacosanol radioactivity in digestive tracts, spleen, kidney, heart, and lung^33^. Further study is required to understand the effect of octacosanol and policosanol in various organs.

Several factors are involved in energy expenditure and thermogenesis in BAT and beige fat^3,13,14^. Policosanol is a fatty alcohol, which is converted to saturated and monounsaturated FAs by fatty aldehyde dehydrogenase (FALDH, alternatively known as Aldh3a2)^42,43^. Fatty aldehydes are also implicated in thyroid function^44^, cell proliferation^45^, adipogenesis and signalling pathways involving PPARγ^46^. In this study, we also observed higher expression of *Aldh3a2* in iWAT of HFDo/p mice and in BAT and iWAT of chow-fed mice treated with octacosanol (Supplementary Fig. S2), suggesting the conversion of octacosanol and policosanol into FAs in BAT and iWAT. Previous studies have suggested that orally administrated octacosanol and policosanol may be oxidised and degraded into shortened saturated and monounsaturated FAs via β-oxidation *in vivo*^33–35^. Thus, another possibility is that octacosanol and policosanol may be degraded to FAs via β-oxidation in liver, which were then transported to BAT and WAT to be utilized for thermogenesis. Additionally, long chain and very long chain saturated and monounsaturated FAs synthesized via Elovl6 and Elovl3 regulate mitochondrial function and thermogenic capacity in BAT and iWAT^38–40,47,48^. Our findings suggest the possibility that shortened saturated and unsaturated FAs metabolized from octacosanol and policosanol perform cellular functions important for thermogenesis and energy expenditure in BAT and WAT. Further studies are needed to confirm this possibility.

Recent studies have shown that FFAR4 (GPR120), a receptor for polyunsaturated FAs (PUFAs), plays a pivotal role in the thermogenic activity of BAT and browning of WAT^49–51^. Activation of FFAR4 by *n*-3 PUFA or a selective agonist increases FA oxidation and reduces BW and body fat by inducing BAT activity and promoting the browning of WAT. In this study, we showed that the treatment of HFDo/p induced *Ffar4* expression in BAT and iWAT of mice and decreased lipid content in BAT and WAT. This suggests that brown/beige fat activation by octacosanol or policosanol could be mediated at least partially through upregulating *Ffar4* expression in BAT and iWAT of HFD-induced mice.

Besides improving metabolism, octacosanol also possesses anti-inflammatory properties^24–26^. In obesity, a paracrine loop between adipocytes and macrophages augments chronic inflammation of adipose tissue, thereby inducing systemic insulin resistance and ectopic lipid accumulation^52^. Since adipose tissue in obese individuals is characterized by adipocyte hypertrophy and chronic inflammation, we also investigated the effects of octacosanol and policosanol on the inflammatory state of WAT. Treatment of HFDo/p lowered the expression levels of pro-inflammatory genes including *F4/80*, *Cd68*, *Tnfa* and *Il1b* in eWAT. Previously, a high concentration of octacosanol has been shown to inhibit the mitogen-activated protein kinase/nuclear factor-kappa B/ activator protein-1 signalling pathway involved in the regulation of inflammation in macrophages^26^. This anti-inflammatory effect of octacosanol and policosanol might contribute to the improvement of systemic insulin resistance induced by HFD. Moreover, octacosanol and policosanol-induced *Ffar4* expression might contribute toward the anti-inflammatory effect of octacosanol and policosanol because FFAR4 activation by *n*-3 PUFA triggers a broad spectrum of anti-inflammatory effects in macrophages^53,54^.

Several studies reported potential serum lipid-lowering effect of octacosanol in rabbits^18^, rats fed an HFD^17^, and hyperlipidemic patients^55,56^. In our experiment using HFD-fed C57BL/6J mice, we were unable to reproduce this benefit. This discrepancy may be due to different dose and duration of administration, animal species, and the degree of hyperlipidemia. In agreement with our result, policosanol treatment failed to lower serum cholesterol in subjects with normal or moderately elevated cholesterol concentrations^19^.

Previous studies have demonstrated that octacosanol and policosanol inhibits cholesterol synthesis in animals^57^ and cultured cells^20,58^. Similar results were obtained in the present study. Octacosanol and policosanol significantly decreased hepatic TC levels accompanied by downregulation of *Ldlr* expression in the liver of HFD-fed mice, suggesting that these lipids suppressed cholesterol uptake in the liver. Also, the downregulation of lipogenic genes by the treatment of octacosanol and policosanol could contribute to reduce hepatic TG accumulation in the liver. We showed that octacosanol and policosanol reduced hepatic TG accumulation in HFD-fed mice by 32% and 18%, respectively. These reductions are equivalent to the estimated the contribution of *de novo* lipid synthesis to hepatic TG content (close to 30% came from *de novo* lipogenesis) in NAFLD patients^59^. It remains unclear if dietary octacosanol and policosanol can affect lipid absorption from the intestine, but one study reported that lipid absorption was not affected by octacosanol in HFD-fed rat^17^.

In conclusion, we show that octacosanol and policosanol exert beneficial metabolic effects by activating thermogenic changes in HFD-induced obesity in mice. Although precise molecular mechanisms underlying the involvement of octacosanol and policosanol in the thermogenic activity of BAT and iWAT remains to be determined, our data shown that octacosanol and policosanol are potent dietary anti-obesity molecules, which increase the thermogenic activity of BAT, thereby increasing energy expenditure and reducing body fat.

## Methods

### Animals and growth conditions

All animal husbandry and animal experiments complied with regulations of the University of Tsukuba for animal experiments and were approved by the Animal Experiment Committee of the University of Tsukuba. Seven-week-old C57BL/6 mice were obtained from CLEA Japan (Tokyo, Japan). Mice were housed in colony cages under a 12 h light/12 h dark cycle, with unlimited access to food and water. At 10 weeks of age, mice were randomly divided into the following four groups: chow group (fed on standard chow with vehicle treatment), HFD group (fed with vehicle treatment), the HFD + octacosanol group (60 mg/kg/day; Sigma-Aldrich, Tokyo, Japan), and HFD + policosanol group (60 mg/kg/day; NOF, Tokyo, Japan). Octacosanol and policosanol were dissolved in 10 mg/ml Acacia-gum water. Octacosanol, policosanol, or the same volume of vehicle was administered via oral gavage once a day for four weeks. At the end of four weeks, all mice were sacrificed in the early light phase in a non-fasting state. HFD was obtained from Oriental Yeast (Tokyo, Japan) and the composition of HFD is shown in Supplementary Table S1. The policosanol extracted from rice bran oil was obtained from NOF (Tokyo, Japan). The composition of aliphatic alcohols in policosanol is C22; 1%, C24; 9%, C26; 9%, C28; 18%, C30; 21%, C32; 9%, C34; 16%, C36; 14%, and others; 3%.

### Dual-energy X-ray absorptiometry (DEXA) analysis

PIXImus2 DEXA (GE Medical Systems Lunar, Madison, WI, USA) was used to measure the weight and percent lean body tissue and fat mass.

### Metabolic measurements

Glucose, insulin, TG, TC and FFA levels in plasma and TG and TC levels in the liver were determined as described previously^60^. Blood samples were taken during the light phase after food deprivation for four hours.

### Body temperature measurement

Core body temperature was measured between 5:00 PM and 6:00 PM using a rectal probe attached to a digital thermometer (RET-3 rectal probe, BRC BDT-100 thermometer; Physitemp, Clifton, NJ).

### Histological analysis

Interscapular BAT (iBAT), iWAT, and eWAT were removed, fixed in 10% buffered formalin, embedded in paraffin, cut into 4 µm thick sections and used for hematoxylin and eosin (H&E) staining and immunohistochemistry as described previously^61^. Immunohistochemical staining for UCP-1 (1:400, ab10983; Abcam, Cambridge, UK) and CD68 (1:400, MCA1957GA; Bio-Rad, Hercules, USA) primary antibodies and detection with secondary antibodies (anti-rabbit IgG, Alexa Fluor 555 conjugate, 1:500, #4413; Cell Signaling Technology Japan, Tokyo, Japan or goat anti-rat IgG, Alexafluor 488 conjugate, 1:500, ab150157; Abcam, Cambridge, UK) was performed as previously described^62^. All images were acquired using a BZ-X710 microscope (Keyence, Osaka, Japan) and data were analysed using a BZ-H3 Analyzer (Keyence, Osaka, Japan). Adipocyte cell size was measured using ImageJ software (National Institutes of Health, Bethesda, MD) and at least 300 cells were counted in each sample.

### Western blotting

Western blotting was performed as previously described^60^. Aliquots of whole cell lysate (30 μg) proteins extracted from BAT or iWAT were loaded onto 10% SDS-PAGE gels and transferred to PDVF membranes (Millipore, Darmstadt, Germany). Membranes were probed with anti-UCP-1 (1:1000, ab10983; Abcam, Cambridge, UK) and anti-GAPDH (1:5000, sc-32233; Santa Cruz Biotechnology, Dallas, Texas, USA) followed by horseradish peroxidase (HRP)-conjugated anti-mouse or anti-rabbit IgG (Cell Signaling Technology Japan, Tokyo, Japan). Immune complexes were visualized using enhanced chemiluminescence (GE Healthcare Japan, Tokyo, Japan).

### RNA extraction and quantitative real-time PCR (qRT-PCR)

Total RNA was extracted from BAT, iWAT, eWAT and liver and used for cDNA synthesis and qRT-PCR as described previously^60–63^. Primer sequences for *Adrb3*, *Aldh3a2*, *Cs*, *Elovl3*, *Fgf21* and *Ffar4* are listed in Supplementary Table S2. The mRNA levels of these genes were normalized relative to cyclophilin mRNA and expressed relative to the appropriate experimental control using the ΔΔCT method.

### Statistical analysis

All data are expressed as the mean ± SEM. Data were compared using Student’s *t*-test (between two groups) or analysis of variance (ANOVA; more than two groups) followed with scheffe post hoc analysis and were considered statistically significant if *p* < 0.05.

## Supplementary information

Supplementary Information
