# Supplementary material for: Octacosanol and policosanol prevent high-fat diet-induced obesity and metabolic disorders by activating brown adipose tissue and improving liver metabolism
Source: Sci Rep. 2019 Mar 26;9:5169. doi: 10.1038/s41598-019-41631-1 (PMC6435753; doi:10.1038/s41598-019-41631-1)
Supplement: Supplementary file 1 — Supplementary Information [file 41598_2019_41631_MOESM1_ESM.pdf]

Supplementary Information

Octacosanol and policosanol prevent high-fat diet-induced obesity and metabolic disorders by activating brown adipose tissue and improving liver metabolism

Rahul Sharma[1], Takashi Matsuzaka[1,2,*], Mahesh K. Kaushik[3], Takehito Sugasawa[1], Hiroshi Ohno[1], Yunong Wang[1], Kaori Motomura[1], Takuya Shimura[1], Yuka Okajima[1], Yuhei Mizunoe[1], Yang Ma[1], Zahara M. Saber[1], Hitoshi Iwasaki[1], Shigeru Yatoh[1], Hiroaki Suzuki[1], Yuichi Aita[1], Song-iee Han[1], Yoshinori Takeuchi[1], Naoya Yahagi[1], Takafumi Miyamoto[1,2], Motohiro Sekiya[1], Yoshimi Nakagawa[1,3] and Hitoshi Shimano[1,3,4,5,*]

[1]Department of Internal Medicine (Endocrinology and Metabolism), Faculty of Medicine, University of Tsukuba, 1-1-1 Tennodai, Tsukuba, Ibaraki 305-8575, Japan

[2]Transborder Medical Research Center, University of Tsukuba, 1-1-1 Tennodai, Tsukuba, Ibaraki 305-8575, Japan

[3]International Institute for Integrative Sleep Medicine (WPI-IIIS), University of Tsukuba, 1-1-1 Tennodai, Tsukuba, Ibaraki 305-8575, Japan

[4]Life Science Center for Survival Dynamics, Tsukuba Advanced Research Alliance (TARA), University of Tsukuba, 1-1-1 Tennodai, Tsukuba, Ibaraki 305-8575, Japan

[5]AMED-CREST, Japan Agency for Medical Research and Development (AMED), 1-7-1, Ohte-machi, Chiyoda-ku, Tokyo, 100-0004, Japan

* Correspondence to:

Takashi Matsuzaka, Ph.D.

Department of Internal Medicine (Endocrinology and Metabolism), Faculty of Medicine, University of Tsukuba, 1-1-1 Tennodai, Tsukuba, Ibaraki 305-8575, Japan

Tel. and Fax: +81-29-853-3174; E-mail: t-matsuz@md.tsukuba.ac.jp

Hitoshi Shimano, MD, Ph.D.

Department of Internal Medicine (Endocrinology and Metabolism), Faculty of Medicine, University of Tsukuba, 1-1-1 Tennodai, Tsukuba, Ibaraki 305-8575, Japan

Tel. and Fax: +81-29-863-2081; E-mail: hshimano@md.tsukuba.ac.jp

Supplementary Table S1.

The composition of the high-fat diet (HFD) used in this study.

Composition	%
Casein	25.0
Corn starch	14.869
Sucrose	20.000
Soybean oil	2.000
Cellurose	5.0
Beef tallow	14.0
Lard	14.0
soybean oil	2.0
Cholesterol	1.250
Cholic acid	0.500
Mineral mix AIN93G	3.5
Vitamin mix AIN93	1.0
Choline bitartrate	0.25
tert-Butylhydroquinone	0.006
L-cystin	0.375
Total	100.0

Supplementary Table S2.

Primer sequence information for the quantitative real-time PCR analysis.

Gene	Forward primer	Reverse primer
Adrb3	CCTTGGGCGAAACTGGTTG	GTTGGTGACAGCTAGGTAGCG
Aldh3a2	TTCTCGTAACAATAAGCTCATCAAACG	CAGCATCCCCAGCCTTCCTTTGTTG
Cs	GGACAATTTTCCAACCAATCTGC	TCGGTTCATTCCCTCTGCATA
Elovl3	TTCTCACGCGGGTTAAAAATGG	GAGCAACAGATAGACGACCAC
Ffar4	ACCAAGTCAATCGCACCCAC	GTGAGACGACAAAGATGAGCC

UCP-1

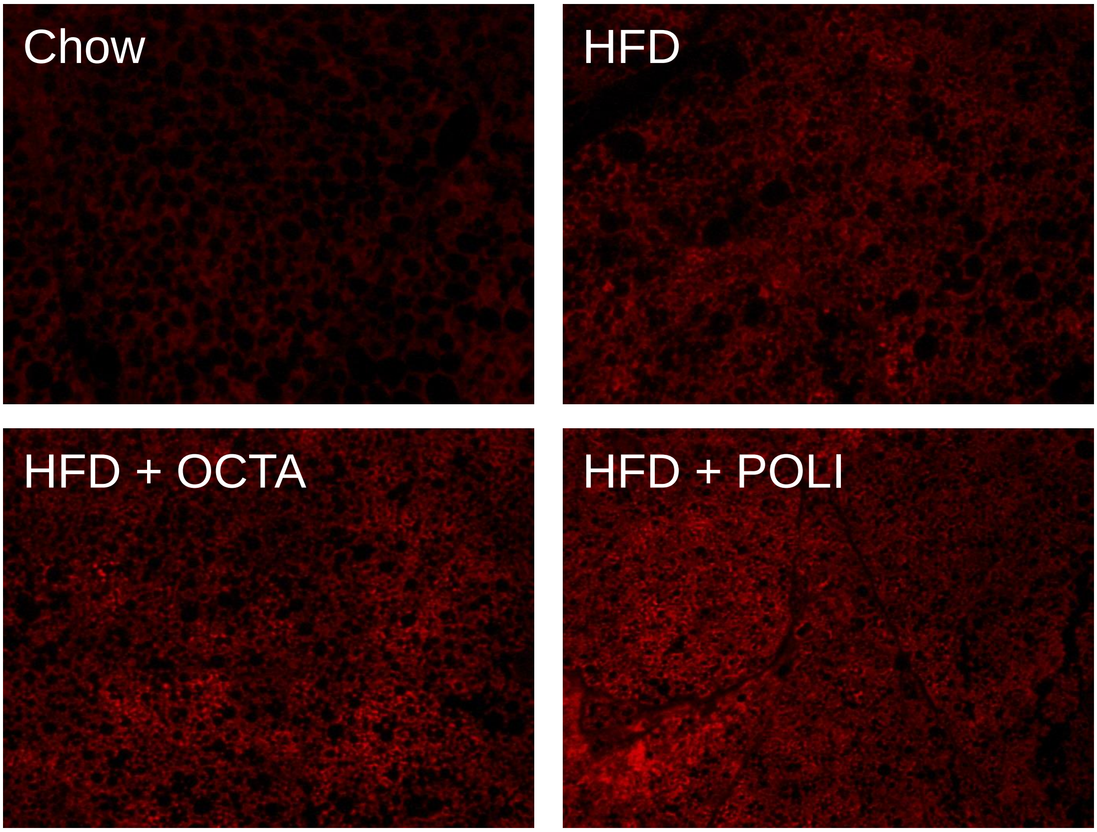

Supplementary Figure S1.

Representative immunohistochemical staining for UCP-1 in BAT sections of mice fed on chow, HFD and HFD treated with octacosanol or policosanol for four weeks.

a

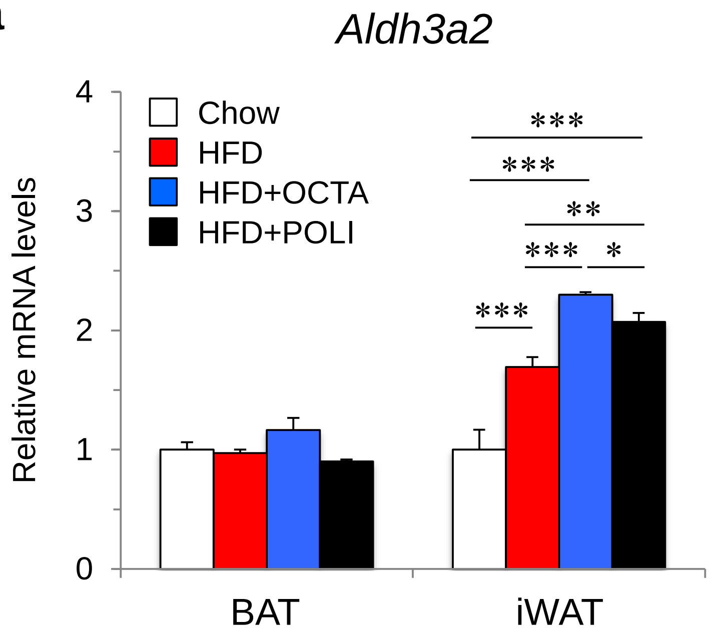
Aldh3a2
Relative mRNA levels
4
3
2
1
0
Chow
HFD
HFD+OCTA
HFD+POLI
***
***
**
***
*
***
BAT
iWAT

b

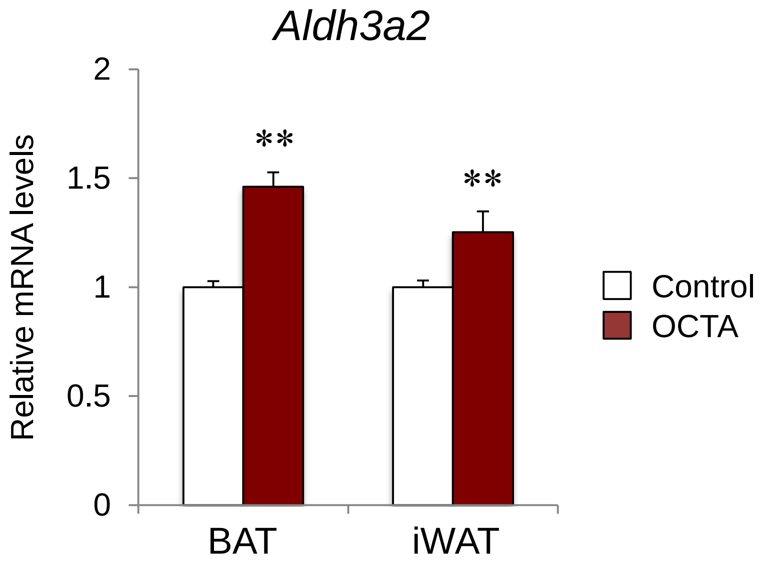
Aldh3a2
Relative mRNA levels
2
1.5
1
0.5
0
**
**
Control
OCTA
BAT
iWAT

Supplementary Figure S2.

Effect of octacosanol and policosanol on the expression of *Aldh3a2* in BAT and iWAT from mice fed a normal chow or HFD.

(**a**) Quantitative real-time PCR analysis of *Aldh3a2* in BAT and iWAT from mice fed on chow, HFD and HFD treated with octacosanol or policosanol for four weeks. Values represent means ± SEM (n = 5–8). * $P < 0.05$, ** $P < 0.01$, *** $P < 0.001$ by using one-way ANOVA followed by scheffe post hoc test. (**b**) Quantitative real-time PCR analysis of *Aldh3a2* in BAT and iWAT from chow-fed mice treated with or without octacosanol for 7 days. Values represent means ± SEM (n = 5). * $P < 0.05$, ** $P < 0.01$, *** $P < 0.001$ vs. control (vehicle-treated) mice by using student t-test.

Figure 3B

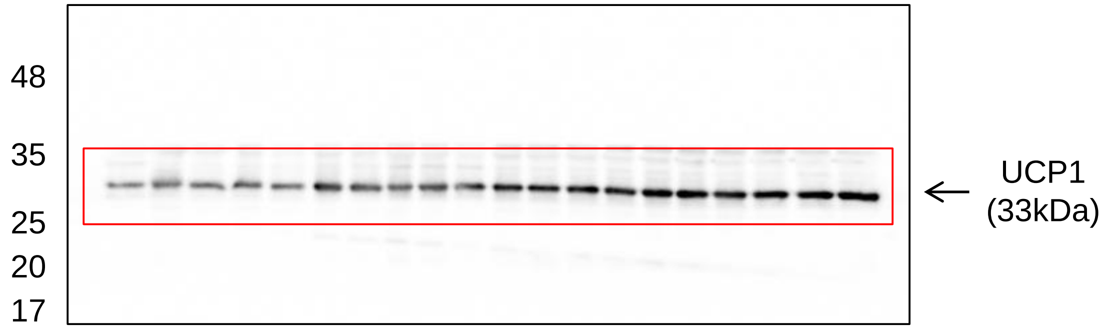

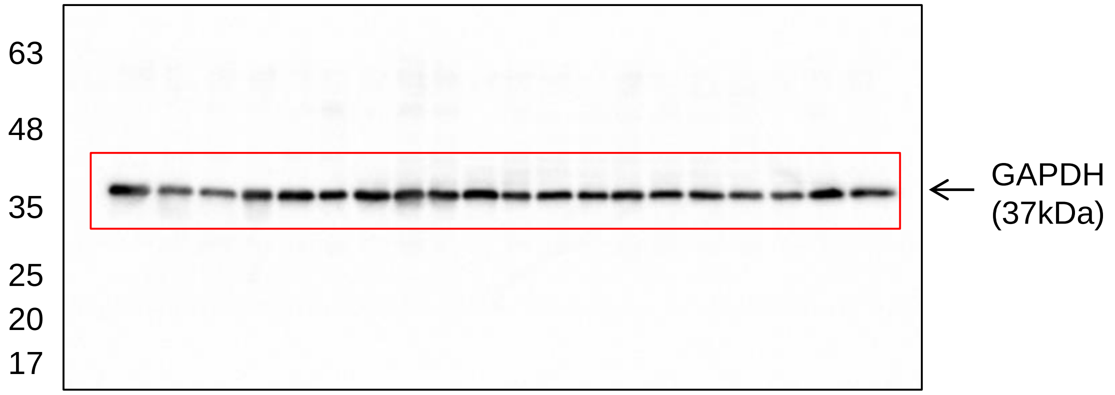

Figure 4C

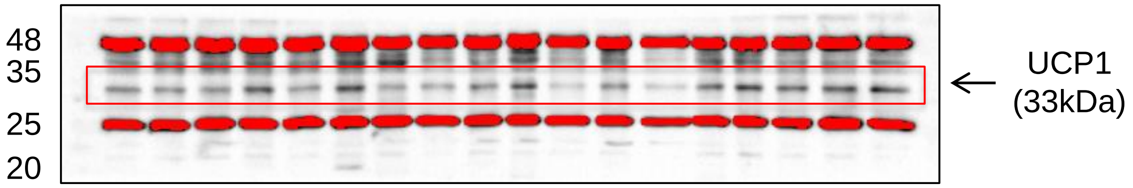

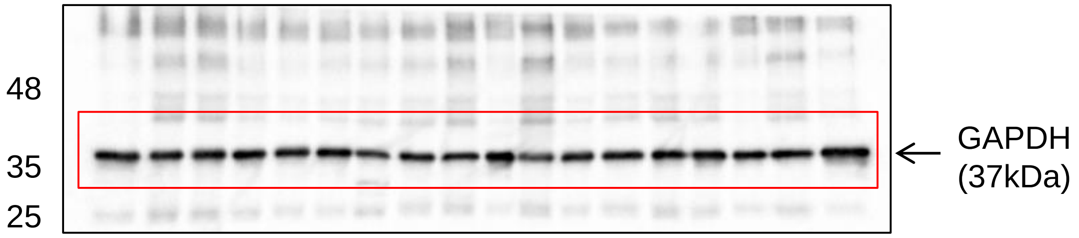

Supplementary Figure S3.

Full scans of Western blots. Specific bands shown in the figures are highlighted by red boxes.
